# Supplementary material for: Machine learning for predicting cognitive decline within five years in Parkinson’s disease: Comparing cognitive assessment scales with DAT SPECT and clinical biomarkers
Source: PLoS One. 2024 Jul 17;19(7):e0304355. doi: 10.1371/journal.pone.0304355 (PMC11253925; doi:10.1371/journal.pone.0304355)
Supplement: S1 File — Consist of three parts 1) S1 Appandix DAT SPECT image characteristics 2) S1 Protocol Image Preprocessing 3) S2 Protocol 3D-Autoencoder. (DOCX) [file pone.0304355.s001.docx]

### **S1 Appandix DAT SPECT image characteristics**

Imaging was performed at screening 4.0 ± 0.5 h after injection of DAT SPECT (123I-Ioflupane; 111-185 MBq) according to the imaging technical operations manual ([www.ppmi-info.org](http://www.ppmi-info.org)) (1). Thyroid update was prevented by pre-treating subjects with either a saturated iodine solution (10 drops in water) or perchlorate (1000 mg) before receiving an injection. The data set included 128 × 128 raw SPECT projection data acquired every 3 degrees, 120 projections, 20% symmetric photopeak windows centered on 159 keV and 122 keV, and a total scan duration of 30-45 minutes. A central core imaging laboratory developed a program for technical qualification, quality assurance, and ongoing camera quality control to ensure technical standardization across multiple sites and cameras used. The same protocol used for PPMI subjects was used to fill an anthropomorphic striatal phantom with 123-I. This phantom was used to test the accuracy and resolution of the reconstructed image volume, as well as to create a site-specific attenuation correction factor (mu) for use with the data at the imaging core lab. The raw projection data was transferred from the sites to the central core imaging laboratory for quality control, which included motion assessment, standardized reconstruction, attenuation correction, and quantification. The raw projection data from a SPECT scan were imported into a HERMES (Hermes Medical Solutions, Skeppsbron 44, 111 30 Stockholm, Sweden) system for iterative (HOSEM) reconstruction. In other words, this was done for all imaging data to ensure that the reconstructions were consistent. Iterative reconstruction was performed with no filtering. The reconstructed HOSEM files were then sent to PMOD (PMOD Technologies, Zurich, Switzerland) for further processing. Attenuation correction ellipses were drawn on the images, and a Chang 0 attenuation correction was applied to images using a site-specific mu derived empirically from phantom data collected during trial site initiation. After that, standard 3D Gaussian post-smoothing (6.0 mm FWHM) was used. Furthermore, to ensure consistent anatomical alignment across scans, these files were normalized to a SPECT ioflupane reference template in standard Montreal Neurologic Institute (MNI) space. The most robust normalizations for the ioflupane DAT-SPECT image volumes were obtained using intramodality spatial normalization using the standard template. The transaxial slice with the highest striatal uptake was then identified, and the eighth hottest striatal slices around it were averaged to produce a single-slice image. Finally, the images collected from the PPMI 1 database share characteristics such as image dimensions (X, Y, Z): 91 × 109 × 91, voxel dimensions (X, Y, Z): 2 × 2 × 2 Millimeters, etc.

**S1 Protocol Image Preprocessing**

For the segmentation of the dorsal striatum on DAT SPECT, we first used four steps as shown in S1 Fig, including (1) an averaging filter to smooth 3D images, (2) a contrast-limited adaptive histogram equalization tool to improve the contrast of the grayscale 3D images, (3) experimentally splitting related pieces, including the right and left Striatum, and then using a threshold (40 percent of maximum intensity) to digitize the image, and then we cropped 3D images based on calculated ROI (4). Finally, we reseized all the images to The final size of 32 X 32 X 32. This, in turn, allows the network to be more robust and efficient in classifying PD patients from healthy controls and also be more efficient. Another preprocessing method we are applying here is intensity normalization (Figure 2). We also augmented images just by flipping them from left to right as suggested in previous studies (2). To maintain dataset size and labels, we double the size of labels and features with no further augmentation.

### **S2 Protocol 3D-Autoencoder**

Deep Learning algorithms can automatically learn features from data, eliminating the need for manual feature engineering. This is important in the field of medical imaging, because there may be many features and interactions that are hard for people to notice. Therefore, this study aimed to predict the outcomes using DFs obtained from autoencoder methods.

A portion of the photos was cropped based on segmentation size before being supplied to autoencoder methods for DF extraction. As shown in S2 Fig an autoencoder consists of an encoder network and a decoder network. The encoder layer transforms the input images into a latent representation or bottleneck, which is then decoded back to the original images. It has four convolutional layers, each with a batch normalization and max-pooling operation. The pooling layers help reduce the number of parameters. The decoder path has four convolutional layers, each with batch normalization. We used a loss function called binary cross-entropy for the proposed autoencoder. We trained the proposed autoencoder using Adam, a gradient-based optimization method, to minimize the loss function. We extracted 1024 DFs from the bottleneck layer using DAT SPECT scans and the 3D autoencoder model.

**References**

1. Marek K, Chowdhury S, Siderowf A, Lasch S, Coffey CS, Caspell-Garcia C, et al. The Parkinson’s progression markers initiative (PPMI)–establishing a PD biomarker cohort. Ann Clin Transl Neurol. 2018;5(12):1460–77.

2. Adams MP, Tang J, Arman,Rahmim. Improved motor outcome prediction in Parkinson’s disease applying deep learning to DaTscan SPECT images. Comput Biol Med. 2021;132:104312.
