# Supplementary figures and images for: Machine learning for predicting cognitive decline within five years in Parkinson’s disease: Comparing cognitive assessment scales with DAT SPECT and clinical biomarkers
Source: PLoS One. 2024 Jul 17;19(7):e0304355. doi: 10.1371/journal.pone.0304355 (PMC11253925; doi:10.1371/journal.pone.0304355)

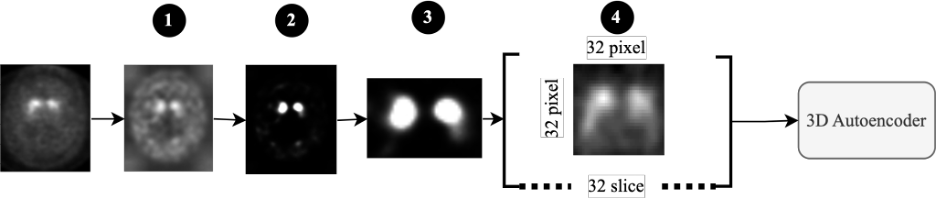

Supplement: S1 Fig — 1) Smooth images 2) Increase the contrast of the images 3) Use a threshold to digitize the image 4) Crop 3D ROI. (TIFF) [file pone.0304355.s002.tiff]

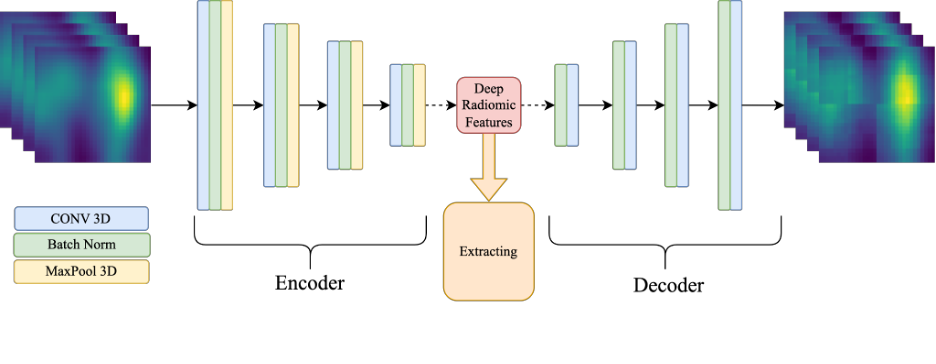

Supplement: S2 Fig — It has four convolutional layers, each followed by a batch normalization and max-pooling operation. The pooling layers are used to reduce the number of parameters. The decoder path has four convolutional layers, each followed by batch normalization. (TIFF) [file pone.0304355.s003.tiff]

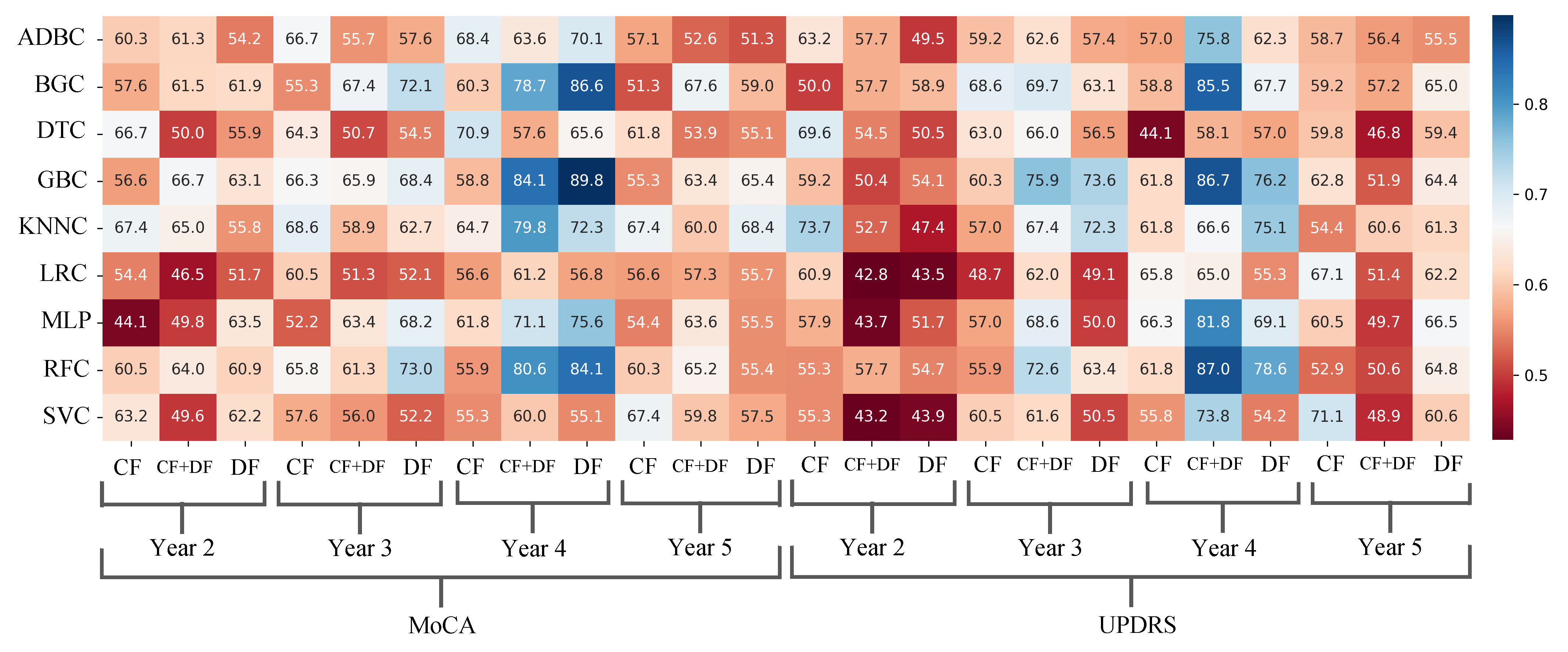

Supplement: S3 Fig — ADBC: AdaBoost Classifier, BGC: Bagging Classifier, SVC: Support Vector Classifier, KNN: K-Nearest Neighbors Classifier, RFC: Random Forest Classifier, GBC: Gradient Boosting Classifier, MLP: Multi-Layer Perceptron, DTC: Decision Tree Classifier, LRC: Logistic Regression Classifier, CF: Clinical Features, DF: Deep Features. (TIFF) [file pone.0304355.s004.tiff]
