# Supplementary material for: Machine learning for predicting cognitive decline within five years in Parkinson’s disease: Comparing cognitive assessment scales with DAT SPECT and clinical biomarkers
Source: PLoS One. 2024 Jul 17;19(7):e0304355. doi: 10.1371/journal.pone.0304355 (PMC11253925; doi:10.1371/journal.pone.0304355)
Supplement: S1 Table — In the MDS-UPDRS-I, we utilized a summation of five sections that assess cognitive-related symptoms in Parkinson’s Disease (PD). The total response to these items is scored on a scale of 0 to 20. Additionally, itemw from the MoCA is referenced in the accompanying table. The total response to these items is scored on a scale of 0 to 30. MDS-UPDRDS-I: The Movement Disorder Society-Unified Parkinson’s Disease Rating Scale, MoCA: Montreal Cognitive Assessment. (DOCX) [file pone.0304355.s005.docx]

**S1 Table. Detail of MDS-UPDRS-I and MoCA questionnaires**

| Questionnaires | Cognitive  related symptoms | Description | |
| --- | --- | --- | --- |
| MDS-UPDRDS-I | COGNITIVE IMPAIRMENT | | 0: Normal: No cognitive impairment.  : Slight: Impairment appreciated by patient or caregiver with no concrete interference with the patient’s ability to carry out normal activities and social interactions.  2: Mild: Clinically evident cognitive dysfunction, but only minimal interference with the patient’s ability to carry out normal activities and social interactions.  3: Moderate: Cognitive deficits interfere with but do not preclude the patient’s ability to carry out normal activities and social interactions.  4: Severe: Cognitive dysfunction precludes the patient’s ability to carry out normal activities and social interactions. |
|  | HALLUCINATIONS AND PSYCHOSIS | | 0: Normal: No hallucinations or psychotic behavior.  1: Slight: Illusions or non-formed hallucinations, but patient recognizes them without loss of  insight.  2: Mild: Formed hallucinations independent of environmental stimuli. No loss of  insight.  3: Moderate: Formed hallucinations with loss of insight.  4: Severe: Patient has delusions or paranoia. |
|  | DEPRESSED MOOD | | 0: Normal: No depressed mood.  1: Slight: Episodes of depressed mood that are not sustained for more than one day at a time. No interference with patient’s ability to carry out normal activities and social interactions.  2: Mild: Depressed mood that is sustained over days, but without interference with normal activities and social interactions.  3: Moderate: Depressed mood that interferes with, but does not preclude the patient’s ability to carry out normal activities and social interactions. 4: Severe: Depressed mood precludes patient’s ability to carry out normal activities and social interactions. |
|  | ANXIOUS MOOD | | 0: Normal: No anxious feelings.  1: Slight: Anxious feelings present but not sustained for more than one day at a time. No interference with patient’s ability to carry out normal activities and social interactions.  2: Mild: Anxious feelings are sustained over more than one day at a time, but without interference with patient’s ability to carry out normal activities and social interactions.  3: Moderate: Anxious feelings interfere with, but do not preclude, the patient’s ability to carry out normal activities and social interactions.  4: Severe: Anxious feelings preclude patient’s ability to carry out normal activities and social interactions. |
|  | APATHY | | 0: Normal: No apathy.  1: Slight: Apathy appreciated by patient and/or caregiver, but no interference with daily activities and social interactions.  2: Mild: Apathy interferes with isolated activities and social interactions.  3: Moderate: Apathy interferes with most activities and social interactions.  4: Severe: Passive and withdrawn, complete loss of initiative. |
| MOCA | VISUOSPATIAL/  EXECUTIVE | | 8 scores: Using a clock-drawing task (3 points) and a three-dimensional cube copy (1 point). Using an alternation task adapted from the trail-making B task (1 point), a phonemic fluency task (1 point), and a two-item verbal abstraction task (2 points). |
|  | MEMORY | | 5 scores: Involves two learning trials of five nouns and delayed recall after approximately five minutes. |
|  | ATTENTION | | 5 scores: Using a sustained attention task (target detection using tapping; 1 point), a serial subtraction task (3 points), and digits forward and backward (1 point each). |
|  | LANGUAGE | | 5 scores: Using a three-item confrontation naming task with low-familiarity animals (lion, camel, rhinoceros; 3 points), repetition of two syntactically complex sentences (2 points), and the aforementioned fluency task. |
|  | ABSTRACTION | | 2 scores: Using a describe-the-similarity task with 2 points being available. |
|  | ORIENTATION | | 6 scores: Evaluated by asking the subject for the date and the city in which the test is occurring (6 points). |

*In the MDS-UPDRS-I, we utilized a summation of five sections that assess cognitive-related symptoms in Parkinson's Disease (PD). The total response to these items is scored on a scale of 0 to 20. Additionally, itemw from the MoCA is referenced in the accompanying table. The total response to these items is scored on a scale of 0 to 30 . MDS-UPDRDS-I :The Movement Disorder Society-Unified Parkinson's Disease Rating Scale, MoCA: Montreal Cognitive Assessment.*
