# Supplementary material for: Machine learning for predicting cognitive decline within five years in Parkinson’s disease: Comparing cognitive assessment scales with DAT SPECT and clinical biomarkers
Source: PLoS One. 2024 Jul 17;19(7):e0304355. doi: 10.1371/journal.pone.0304355 (PMC11253925; doi:10.1371/journal.pone.0304355)
Supplement: S2 Table — In year 2, there was a significant gender-related discrepancy observed between the PD-CD and PD-NC groups. Year 3 highlighted a substantial difference in PIGD scores between the same groups. However, in years 4 and 5, the analysis revealed no significant differences between PD-CD and PD-NC. (DOCX) [file pone.0304355.s006.docx]

| Feature | MoCA - Year 2 | | | MoCA - Year 3 | | | MoCA - Year 4 | | | MoCA - Year 5 | | |
| --- | --- | --- | --- | --- | --- | --- | --- | --- | --- | --- | --- | --- |
|  | PD-CD | PD-NC | p-value | PD-CD | PD-NC | p-value | PD-CD | PD-NC | p-value | PD-CD | PD-NC | p-value |
|  | n=151 | n=179 |  | n=153 | n=177 |  | n=102 | n=228 |  | n=142 | n=188 |  |
| Age | 61.51 | 60.95 | 0.6004 | 62.03 | 60.49 | 0.1469 | 62.02 | 60.84 | 0.3050 | 60.78 | 61.52 | 0.4861 |
| amyloid-β 42 | 889.85 | 917.91 | 0.5067 | 899.33 | 910.03 | 0.7998 | 880.40 | 916.10 | 0.4333 | 891.31 | 915.46 | 0.5701 |
| α-Synuclein | 1447.03 | 1528.20 | 0.2615 | 1508.43 | 1476.04 | 0.6541 | 1443.21 | 1512.46 | 0.3744 | 1406.20 | 1555.15 | 0.0401 * |
| Hallucination | 0.05 | 0.04 | 0.7683 | 0.07 | 0.03 | 0.1197 | 0.04 | 0.05 | 0.6581 | 0.06 | 0.04 | 0.6262 |
| MDS-UPDRS-III | 20.60 | 19.77 | 0.3945 | 20.42 | 19.92 | 0.6103 | 21.17 | 19.70 | 0.1625 | 21.17 | 19.38 | 0.0686 |
| PIDG | 1.34 | 1.31 | 0.5953 | 1.32 | 1.33 | 0.9810 | 1.28 | 1.34 | 0.2823 | 1.31 | 1.33 | 0.7142 |
| P-tau | 13.00 | 14.21 | 0.0505 | 13.70 | 13.62 | 0.9018 | 13.54 | 13.71 | 0.7942 | 12.91 | 14.22 | 0.0348 * |
| T-tau | 154.82 | 168.96 | 0.028 * | 162.28 | 162.68 | 0.9503 | 159.20 | 163.96 | 0.4943 | 155.15 | 168.03 | 0.04 * |
| Blood uric acid | 306.25 | 312.76 | 0.4334 | 313.88 | 306.25 | 0.3576 | 318.83 | 305.74 | 0.1436 | 313.79 | 306.76 | 0.4010 |
| Disease duration | 3142.06 | 3179.99 | 0.4736 | 3144.85 | 3178.00 | 0.5307 | 3121.16 | 3181.19 | 0.2925 | 3131.18 | 3186.39 | 0.2997 |
| SCOPA-AUT | 8.36 | 9.21 | 0.2358 | 8.41 | 9.18 | 0.2823 | 9.67 | 8.44 | 0.1099 | 8.96 | 8.72 | 0.7377 |
| GDS | 5.38 | 5.37 | 0.9329 | 5.24 | 5.50 | 0.1512 | 5.52 | 5.31 | 0.2906 | 5.36 | 5.39 | 0.8742 |
| APOE | 2.91 | 2.90 | 0.8962 | 2.92 | 2.89 | 0.7942 | 2.90 | 2.91 | 0.9604 | 2.95 | 2.87 | 0.4825 |
| Gender | 0.63 | 0.61 | 0.7077 | 0.70 | 0.55 | 0.004 * | 0.63 | 0.61 | 0.8174 | 0.68 | 0.57 | 0.0603 |
| Orthostatic hypotension | 0.02 | 0.01 | 0.2388 | 0.01 | 0.01 | 0.8838 | 0.01 | 0.01 | 0.7977 | 0.00 | 0.02 | 0.0808 |
| Diabetes | 0.04 | 0.05 | 0.6481 | 0.06 | 0.03 | 0.2798 | 0.06 | 0.04 | 0.4370 | 0.08 | 0.02 | 0.01 * |
| Hypertension | 0.26 | 0.21 | 0.2690 | 0.26 | 0.20 | 0.2129 | 0.23 | 0.23 | 0.8899 | 0.25 | 0.22 | 0.5456 |

**S2 Table. Results of the paired T-test for the MoCA score.**

*In year 2, a notable difference was observed in T-tau levels between the PD-CD and PD-NC groups. Year 3 saw a significant disparity in gender distribution between the two groups. No substantial differences were detected in year 4. However, in year 5, significant variations were found in a-synuclein, P-tau, T-tau, and diabetes occurrences between the PD-CD and PD-NC groups.*
