# Supplementary material for: Machine learning for predicting cognitive decline within five years in Parkinson’s disease: Comparing cognitive assessment scales with DAT SPECT and clinical biomarkers
Source: PLoS One. 2024 Jul 17;19(7):e0304355. doi: 10.1371/journal.pone.0304355 (PMC11253925; doi:10.1371/journal.pone.0304355)
Supplement: S3 Table — In year 2, a notable difference was observed in T-tau levels between the PD-CD and PD-NC groups. Year 3 saw a significant disparity in gender distribution between the two groups. No substantial differences were detected in year 4. However, in year 5, significant variations were found in a-synuclein, P-tau, T-tau, and diabetes occurrences between the PD-CD and PD-NC groups. (DOCX) [file pone.0304355.s007.docx]

**S3 Table: Paired T-test outcomes for the MDS-UPDRS-I score.**

| Feature | MDS-UPDRS-I - Year 2 | | | MDS-UPDRS-I - Year 3 | | | MDS-UPDRS-I - Year 4 | | | MDS-UPDRS-I - Year 5 | | |
| --- | --- | --- | --- | --- | --- | --- | --- | --- | --- | --- | --- | --- |
|  | PD-CD | PD-NC | p-value | PD-CD | PD-NC | p-value | PD-CD | PD-NC | p-value | PD-CD | PD-NC | p-value |
|  | n=159 | n=171 |  | n=140 | n=190 |  | n=115 | n=215 |  | n=158 | n=172 |  |
| Age | 61.72 | 60.72 | 0.3439 | 61.83 | 60.74 | 0.3104 | 61.10 | 61.26 | 0.8849 | 60.69 | 61.68 | 0.3499 |
| amyloid-β 42 | 908.12 | 902.23 | 0.8889 | 876.81 | 925.89 | 0.2489 | 911.58 | 901.59 | 0.8211 | 895.34 | 914.00 | 0.6581 |
| α-Synuclein | 1498.28 | 1484.35 | 0.8469 | 1473.94 | 1503.68 | 0.6835 | 1397.21 | 1541.26 | 0.0562 | 1425.33 | 1551.43 | 0.0799 |
| Hallucination | 0.06 | 0.04 | 0.5763 | 0.08 | 0.03 | 0.0646 | 0.06 | 0.04 | 0.5179 | 0.04 | 0.06 | 0.4721 |
| MDS-UPDRS-III | 20.48 | 19.85 | 0.5177 | 20.38 | 19.98 | 0.6889 | 20.75 | 19.83 | 0.3701 | 20.89 | 19.48 | 0.1475 |
| PIDG | 1.34 | 1.31 | 0.4825 | 1.38 | 1.28 | 0.0499 * | 1.33 | 1.32 | 0.8810 | 1.33 | 1.32 | 0.8817 |
| P-tau | 13.47 | 13.83 | 0.5601 | 13.62 | 13.68 | 0.9241 | 13.30 | 13.85 | 0.3995 | 13.08 | 14.18 | 0.0742 |
| T-tau | 160.04 | 164.77 | 0.4631 | 161.61 | 163.14 | 0.8151 | 159.44 | 164.12 | 0.4879 | 156.66 | 167.85 | 0.0814 |
| Blood uric acid | 312.17 | 307.57 | 0.5792 | 310.95 | 308.93 | 0.8096 | 315.01 | 306.99 | 0.3555 | 316.64 | 303.49 | 0.1120 |
| Disease duration | 3124.73 | 3197.88 | 0.1652 | 3181.01 | 3149.09 | 0.5497 | 3162.39 | 3162.76 | 0.9946 | 3131.03 | 3191.66 | 0.2504 |
| SCOPA-AUT | 8.71 | 8.92 | 0.7636 | 8.66 | 8.94 | 0.6910 | 9.30 | 8.56 | 0.3184 | 8.85 | 8.80 | 0.9420 |
| GDS | 5.32 | 5.43 | 0.5604 | 5.37 | 5.38 | 0.9675 | 5.26 | 5.44 | 0.3562 | 5.42 | 5.34 | 0.6589 |
| APOE | 2.92 | 2.89 | 0.8317 | 2.86 | 2.94 | 0.4472 | 2.93 | 2.89 | 0.7470 | 2.89 | 2.92 | 0.8128 |
| Gender | 0.67 | 0.57 | 0.0484 * | 0.67 | 0.58 | 0.0879 | 0.69 | 0.58 | 0.0603 | 0.65 | 0.59 | 0.3278 |
| Orthostatic hypotension | 0.01 | 0.01 | 0.9419 | 0.02 | 0.01 | 0.1858 | 0.00 | 0.02 | 0.1420 | 0.01 | 0.01 | 0.9322 |
| Diabetes | 0.07 | 0.02 | 0.0462 | 0.06 | 0.04 | 0.3831 | 0.04 | 0.05 | 0.9001 | 0.05 | 0.04 | 0.6663 |
| Hypertension | 0.23 | 0.23 | 0.9207 | 0.27 | 0.20 | 0.1285 | 0.19 | 0.25 | 0.2197 | 0.27 | 0.19 | 0.0840 |

In year 2, there was a significant gender-related discrepancy observed between the PD-CD and PD-NC groups. Year 3 highlighted a substantial difference in PIGD scores between the same groups. However, in years 4 and 5, the analysis revealed no significant differences between PD-CD and PD-NC.
